# Editorial: Recent innovation in breast reconstructive surgery

**DOI:** 10.3389/fsurg.2023.1135833

**Published:** 2023-02-06

**Authors:** Andrea Lisa, Andrea Battistini, Valeriano Vinci, Mauro Barone

**Affiliations:** ^1^ Humanitas Cancer Center, Humanitas Research Hospital, Rozzano, Italy; ^2^ Campus Bio-Medico University, Rome, Lazio, Italy

**Keywords:** prepectoral breast reconstruction, acellular derma matrix, autologous fat graft, breast reduction, breast implant, nipple sparing mastectomy (NSM)

Editorial on the Research TopicRecent innovation in breast reconstructive surgery

The important role of mastectomy in the advanced stages of breast cancer has led to a growing demand for breast reconstruction. For patients with breast cancer undergoing a mastectomy, preserving the breast mound with immediate or delayed reconstruction is a crucial part to ensure an adequate quality of life (QoL).

For women who opt for breast reconstruction surgery, two main considerations must be made: the type and the timing of reconstruction. To date, several reconstruction techniques are available: two-stage tissue expander and implant (TE/I), single-stage direct-to-implant reconstruction (DTI), and autologous tissue reconstruction (ATR). Breast reconstruction can be performed either at the time of mastectomy (immediate reconstruction) or later (delayed reconstruction). Choosing the optimum type of reconstruction is challenging since many factors come into play. Among them, there are the patient's preferences, the balance of risks and benefits of each technique, the baseline risk factors for reconstruction failure such as high BMI or smoking, and the need for postmastectomy radiotherapy.

Mastectomy techniques have evolved from more radical treatments with the routine removal of the nipple-areolar complex (NAC) to less extensive procedures such as nipple-sparing mastectomies. To further reduce the impact on breast tissue a new modification of nipple-sparing mastectomy, consisting of preservation of the anterior lamellar fat layer, has been proposed. Results described in our special topic are promising since such preservation allows to obtain a thicker flap, thus lowering the complication rate, in particular ischemia of the mastectomy flaps and nipple-areolar complex. At the same time, it leads to a better aesthetic outcome and improved quality of life (Bakhtiyor Najmiddinov et al.).

Nowadays techniques are rapidly evolving to provide the best in terms of quality of reconstruction and fast recovery. In particular, surgeons should consider the effect of adjuvant treatment and in particular radiotherapy's effect on breast reconstruction. Several procedures are adopted to reduce its impact such as the use of autologous tissues (free flaps or pedicled flaps) and fat grafting. Nevertheless, the scientific consensus is still missing.

The new trend in prosthetic reconstruction is represented by prepectoral implant positioning. Such a procedure prevents animation deformity and allows a faster hospital discharge. On the other hand, a good mastectomy flap viability is needed and the complete correlation with adjuvant oncological treatment should be fully understood. Prepectoral reconstruction has been favored by the introduction of biological membranes which have considerably reduced the incidence of capsular contraction, the most severe complication of prepectoral reconstruction in its early stages.

Reconstructive outcomes with ADM have been widely described in the literature, even in the long-term and large cohorts, proving enhanced aesthetic and functional results, improved QoL, and cost-effectiveness compared to submuscular approaches.

Few articles in the literature compare the prepectoral approach with ADM to submuscular plane reconstruction. Such comparison is paramount to guide the physician in the decision-making process, favoring the prepectoral approach if possible. In our special topic, we present a retrospective comparison between patients treated with submuscular reconstruction, prepectoral reconstruction alone, and prepectoral reconstruction with ADM. Patients were evaluated and compared for postoperative pain, overall complication rate, and aesthetic results. Data revealed that prepectoral breast reconstruction with ADM is better than the two techniques (Francesco Klinger et al.).

Such a paper was the first to adopt a new matrix, Fortiva®, as ADM in prepectoral reconstruction, with encouraging results.

Prepectoral breast reconstruction is the new frontier in breast reconstruction, in a continuous effort to obtain better results.

New materials are under evaluation, to improve safety, reduce complication rate, and speed up surgical procedures. One of these is BRAXON®*Fast*, which consists of a ready-to-use ADM. The lens-shaped conformation of the anterior surface easily adapts to the breast implant without the need for tailoring. The surgical procedure is faster since the implant is rapidly and easily inserted into the ADM shell, and by suturing the two ADM flaps together. The preliminary report presented in our special issue confirms that it speeds up the implant wrapping process without multiple intricate and time-consuming wire-passing operations characterizing other devices that could put sterility at stake (Francesco Klinger et al.).

Nevertheless breast reduction represents an essential aspect of breast reconstruction technique for obtaining symmetrization in the case of monolateral mastectomy. Breast reduction is particularly challenging in case of big-size reduction and in obese patients.

The main complication of breast reduction is nipple areola complex necrosis due to vascular compromise.

McKissock vertical bipedicle has been described as a valid procedure to reduce complications. The main benefit introduced was the inclusion of perforators from the superior and inferior poles of the breast.

In our special topic, an interesting case series of 251 patients treated with McKissock reduction mammaplasty is presented and the results are encouraging. Additionally, we discovered no evidence of a statistically significant difference between the subtypes of complications in either group, and our findings are consistent with the rates published in the literature, supporting the non-inferiority of the McKissock approach over the alternatives (Francesco Messana et al.).

In conclusion, our special topic covers different aspects of breast reconstruction and cutting-edge topics that could be of benefit to a dedicated breast reconstructive surgeon.

## Author contributions

All authors contributed to the article and approved the submitted version.

## Conflict of interest

The authors declare that the research was conducted in the absence of any commercial or financial relationships that could be construed as a potential conflict of interest.

## Publisher's note

All claims expressed in this article are solely those of the authors and do not necessarily represent those of their affiliated organizations, or those of the publisher, the editors and the reviewers. Any product that may be evaluated in this article, or claim that may be made by its manufacturer, is not guaranteed or endorsed by the publisher.

